# Phytochemistry, Biological, and Toxicity Study on Aqueous and Methanol Extracts of *Chromolaena odorata*

**DOI:** 10.1155/2023/6689271

**Published:** 2023-10-09

**Authors:** Akash Budha Magar, Deepa Shrestha, Sangita Pakka, Khaga Raj Sharma

**Affiliations:** Central Department of Chemistry, Tribhuvan University, Kirtipur, Kathmandu, Nepal

## Abstract

The medicinal plant *Chromolaena odorata* is traditionally used by people living in different communities of Nepal and the globe against diabetes, soft tissue wounds, skin infections, diarrhea, malaria, and several other infectious diseases. The present study focuses on the qualitative and quantitative phytochemical analyses and antioxidant, antidiabetic, antibacterial, and toxicity of the plant for assessing its pharmacological potential. The extracts of flowers, leaves, and stems were prepared using methanol and distilled water as the extracting solvents. Total phenolic content (TPC) and total flavonoid content (TFC) were estimated by using the Folin–Ciocalteu phenol reagent method and the aluminum chloride colorimetric method. Antioxidant and antidiabetic activities were assessed using the DPPH assay and *α*-glucosidase inhibition assay. A brine shrimp assay was performed to study the toxicity, and the antibacterial activity test was performed by the agar well diffusion method. Phytochemical analysis revealed the presence of phenols, flavonoids, quinones, terpenoids, and coumarins as secondary metabolites. The methanol extract of leaves and flowers displayed the highest phenolic and flavonoid content with 182.26 ± 1.99 mg GAE/g, 128.57 ± 7.62 mg QE/g and 172.65 ± 0.48 mg GAE/g, 121.74 ± 7.06 mg QE/g, respectively. The crude extracts showed the highest DPPH free radical scavenging activity with half maximal inhibitory concentration (IC_50_) of 32.81 ± 5.26 *µ*g/mL and 41.00 ± 1.10 *µ*g/mL, respectively. The methanol extract of the leaves was found to be effective against bacterial strains such as *K. pneumoniae* (ZOI = 9.67 ± 0.32 mm), *B. subtilis* (ZOI = 15.00 ± 0 mm), and *E. coli* (7.3 ± 0.32 mm). The methanol extract of the flowers showed the most *α*-glucosidase inhibitory activity (IC_50_ 227.63 ± 11.38 *µ*g/mL), followed by the methanol extract of leaves (IC_50_ 249.50 ± 0.97 *µ*g/mL). The aqueous extract of the flowers showed the toxic effect with LC_50_ 107.31 ± 49.04 *µ*g/mL against the brine shrimp nauplii. In conclusion, *C. odorata* was found to be a rich source of plant secondary metabolites such as phenolics and flavonoids with potential effects against bacterial infection, diabetes, and oxidative stress in humans. The toxicity study showed that the aqueous extract of flowers possesses pharmacological activities. This study supports the traditional use of the plant against infectious diseases and diabetes and provides some scientific validation.

## 1. Introduction

Diabetes mellitus is a chronic disease caused due to metabolic disorder and is noticed by the high blood sugar in the human body. It is a global health problem affecting an estimated 536.6 million people worldwide as mentioned by the International Diabetes Federation [1]. Type 1 diabetes is caused by immune-associated destruction of pancreatic beta cells, whereas type 2 diabetes results from beta cell dysfunction and insulin resistance [2]. It can lead to cardiovascular and microvascular complications such as retinopathy, neuropathy, and nephropathy [3]. Studies have reported hyperglycemia-associated production of high levels of reactive oxygen species that ultimately leads to vascular damage [4]. Diabetes can be managed by insulin injection, changes in lifestyle, diet, and medication. The synthetic antidiabetic drugs available in the modern world are causing several side effects besides lowering the blood glucose level in human beings. Hence, the search of plant-based natural antidiabetic drug candidates or drugs is an urgent need in modern research. The natural compounds isolated from the plants such as apigenin, curcumin, naringenin, and resveratrol exhibit antihyperglycemic activities showing least side effects with high efficacy [5].

Reactive oxygen species (ROS) including superoxide anion radicals, hydrogen peroxide, and hydroxyl radicals are produced in mitochondria during aerobic respiration [6]. They can cause structural damage to DNA, protein, carbohydrates, lipids, and enzymes that lead to inflammation, age-related cancer, and neurodegeneration [7]. Various enzymatic and nonenzymatic antioxidants neutralize reactive oxygen species and protect our bodies from oxidative damage. Primary and secondary plant metabolites such as vitamin C, vitamin E, polyphenols, flavonoids, and carotenoids play a significant role as natural antioxidants [8]. The rise of multidrug-resistant bacteria that are resistant to almost all available antibiotics has resulted in the need to find noble antimicrobial compounds [9]. Plant secondary metabolites such as polyphenols, alkaloids, terpenoids, coumarins, and essential oils display significant antimicrobial activity [10]. Thus, medicinal plants may act as sources of compounds with therapeutic potential [10]. The study of toxicity in plants can be carried out by the brine shrimp lethality assay which is a cheap, simple, and effective method to report toxic bioactive compounds. In this way, plant secondary metabolites pose a variety of medicinal properties. Plant-based medicine makes up about 25% of the global market in modern medicinal practices. Hence, there is a need for an extensive study into medicinal plants and the incorporation of traditional knowledge with modern science in a country like Nepal which is rich in medicinal plants.

**Figure 1:**
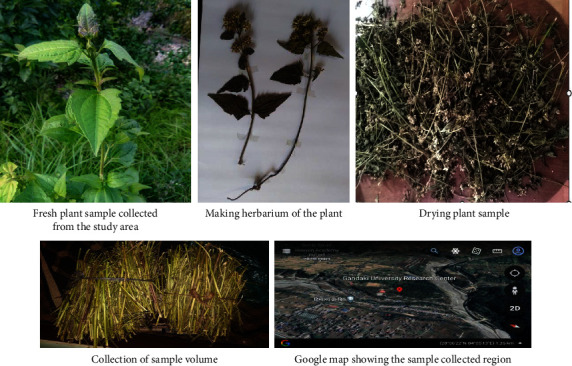
Photographs of plant samples collected from the study area.

*Chromolaena odorata* (L.) King and Robinson (also *Eupatorium odoratum*) is a weedy shrub that belongs to the Asteraceae family. It is native to Central and South America and found in most of the tropical and subtropical habitats around the world including Asia, Africa, and Australia [11, 12]. *C. odorata* is a rapidly growing, scrambling, perennial shrub. Its leaf and flower give off a pungent odor upon being crushed. Despite being an invasive species that can potentially cause harm to local vegetation, *C. odorata* has some beneficial effects in the area of agriculture and medicine [13]. From a previous study, it has been reported that the plant is used in the treatment of wounds, skin infections, malaria, diarrhea, cough, and diabetes [14–16]. It has also been found that the plant shows anti-inflammatory, antipyretic, analgesic, antimicrobial, antioxidant, antihyperglycemic, cytotoxic, and antispasmodic properties [17]. The present study focuses on qualitative phytochemical analysis, estimation of phenolic and flavonoid contents, antioxidant, antidiabetic, and antibacterial activities, and toxicity of the methanol and aqueous plant extracts. To the best of our knowledge, this is the first report on this plant which is growing in the western part of Nepal (Figure 1).

## 2. Materials and Methods

### 2.1. Plant Collection, Identification, and Extract Preparation

The flowers, leaves, and stems of *C. odorata* were harvested from the Kaski district of Nepal in September 2021. The herbarium of the plant was submitted to the Central Department of Botany, Tribhuvan University, Kathmandu, Nepal, for identification of the plant. The identification number was TUCH21052. The harvested plant materials were cleaned, shade-dried, and ground. 300 g of each plant powder was kept in the conical flasks in which 500 mL of methanol was added in one conical flask, whereas the same amount of distilled water was added to another conical flask as the extracting solvent. The flasks were kept for percolation with continuous shaking. After 24 hours, the contents of the conical flask with distilled water were filtered using a clean muslin cloth. The residue was resuspended in the same conical flask with some additional distilled water. The filtrate was again filtered using Whatman filter paper 1 and concentrated in a rotatory evaporator at 40°C. The same process was repeated three times for the complete extraction of the water-soluble metabolites. A similar process was carried out for methanol solvent at intervals of 72, 48, and 24 hours. The crude aqueous and methanol extracts were stored at 4°C to proceed with the biological study and chemical analysis (Table 1).

### 2.2. Phytochemical Analysis

The qualitative preliminary phytochemical analysis of the plant extract was performed by adopting standard protocols [19, 20].

#### 2.2.1. Alkaloids

The plant extract (5 mL) was concentrated to yield a residue. Thus, the obtained residue was dissolved in 1.5 mL of 2% (v/v) HCl. 3 drops of Meyer's reagent were added. The appearance of a white precipitate indicates the presence of alkaloids.

#### 2.2.2. Phenols

The plant extract was mixed with 2 mL of 2% FeCl_3_ solution. The formation of blue-green or black coloration indicates the presence of phenols.

#### 2.2.3. Flavonoids

The plant extract was mixed with a few pieces of magnesium ribbon, and then, conc. HCl was added in a dropwise manner. The formation of a pink scarlet color after a few minutes indicates the presence of flavonoids.

#### 2.2.4. Tannins

2 mL of 5% FeCl_3_ was added to 2 mL of plant extract. The formation of a yellow or brown precipitate indicates the presence of tannins.

#### 2.2.5. Reducing Sugars

0.5 g of plant extract was added to 2.5 mL of Benedict's solution taken in a test tube and then warmed in a hot waterbath for about 5 minutes. The formation of green/red or yellow coloration indicates the presence of reducing sugar.

#### 2.2.6. Saponins

The plant extract was mixed with 5 mL of distilled water and then shaken vigorously. The formation of stable foam indicates the presence of saponins.

#### 2.2.7. Coumarins

A single pellet of KOH was dissolved in 1 mL of ethanol. Then, 1 mL of extract solution was added. The formation of precipitates indicates the presence of coumarins.

#### 2.2.8. Terpenoids

A small amount of plant extract was dissolved in chloroform, and an equal volume of conc. H_2_SO_4_ was added. The formation of a brown ring at the junction of two liquids indicates the presence of terpenoids.

#### 2.2.9. Quinones

1 mL of conc. H_2_SO_4_ was added to 1 mL of plant extract solution and then observed for the formation of red coloration.

#### 2.2.10. Sterols

2 mL of methanol extract of the plant was mixed with chloroform followed by adding 1-2 mL of acetic anhydride. 1 or 2 drops of conc. H_2_SO_4_ was added from the side of the test tube. An array of red, blue, and green colors indicates the presence of sterols.

#### 2.2.11. Glycosides

2 mL of glacial acetic acid and one drop each of 5% FeCl_3_ and conc. H_2_SO_4_ were added to 5 mL of plant extract. The formation of a brown ring indicates the presence of glycosides.

#### 2.2.12. Proteins

The plant extract was mixed with 2 mL of Millon's reagent. The formation of a white precipitate that turns red on gentle heating indicates the presence of proteins.

### 2.3. Total Phenolic Content (TPC)

The total phenolic content of plant extracts was measured by using the Folin–Ciocalteu phenol (FCR) reagent method with slight modifications [21]. A set of gallic acid solutions with concentrations 10, 20, 30, 40, 50, 60, 70, 80, 90, and 100 *µ*g/mL in methanol and 5 mg/mL plant extract solution in 50% DMSO were prepared. 20 *µ*L each of gallic acid and different plant extract solutions were filled in different bores of a 96-well plate in triplicates. 100 *µ*L of FCR (1 : 10 v/v diluted with distilled water) and 80 *µ*L of Na_2_CO_3_ (1 M) were added to each bore. The reaction mixture was placed in the dark for 25 minutes, and then, absorbance was measured at 760 nm by using a microplate reader (SYNERGYILX, BioTek Instruments. Inc., USA). A standard calibration curve was constructed using the absorbance against the different concentrations of gallic acid. TPC of plant extracts was calculated using the standard calibration curve and expressed in terms of milligrams of gallic acid equivalent per gram dry weight of plant extract (mg GAE/g).

### 2.4. Total Flavonoid Content (TFC)

The total flavonoid content of the plant extract was measured by using the aluminum chloride colorimetric method with slight changes [22]. A set of quercetin solutions of concentrations 15.4, 30.8, 46.2, 61.6, 77, 92.4, 107.8, 123.2, 138.6, and 154 *µ*g/mL were prepared in methanol. 130 *µ*L quercetin solution, 60 *µ*L of ethanol, 5 *µ*L of AlCl_3_, and 5 *µ*L of CH_3_COOK were filled in the bores of the 96-well plates in triplicates. Similarly, 20 *µ*L of plant extract (5 mg/mL in 50% DMSO), 110 *µ*L of distilled water, 60 *µ*L of ethanol, 5 *µ*L of AlCl_3_, and 5 *µ*L of CH_3_COOK were added in triplicates to the remaining bores. The reaction mixture was allowed to stand for 30 minutes in the dark, and then, absorbance was measured at 415 nm using a microplate reader. A standard calibration curve was constructed using absorbances of quercetin solutions. TFC of plant extracts was calculated by using the calibration curve constructed, and the values were expressed as milligrams of quercetin equivalent per gram dry weight of plant extract (mg QE/g).

### 2.5. Antioxidant Assay

2, 2-Diphenyl-1-picrylhydrazyl (DPPH) assay was used to evaluate the free radical scavenging activity of plant extracts [23]. Plant extract solutions of 500, 250, 125, 62.5, 31.25, and 15.625 *µ*g/mL were prepared by serial dilution of stock solution (5 mg/mL in 50% DMSO). 100 *µ*L of DPPH solution (0.1 mM in ethanol) and 100 *µ*L of plant extract solution were filled in the bores of a microplate in triplicates. The reaction mixture was placed in the dark for 30 minutes, and then, absorbance was measured at 517 nm. 50% DMSO was used as control, and quercetin was used as standard. The percentage of free radical scavenging was calculated by using the following equation:

(1) $$\begin{array}{c} \text{Percentage scavenging}=\frac{\text{Absorbance of control}-\text{Absorbance of sample}}{\text{Absorbance of control}}\times 100. \end{array}$$

Half maximal inhibitory concentration (IC_50_) was calculated from the plot of percentage scavenging against concentrations of plant extract using GraphPad Prism 9 software.

### 2.6. Antidiabetic Assay

The antidiabetic activity of plant extracts was estimated by using an *α*-glucosidase enzyme inhibition assay [24]. Plant extract solutions of 1000, 500, 250, and 125 *µ*g/mL concentrations were prepared by diluting stock solution (5 mg/mL in 30% DMSO). 20 *µ*L of *α*-glucosidase enzyme (0.5 unit/mL) was premixed with 20 *µ*L of plant extract solution. Then, 120 *µ*L of potassium phosphate buffer (pH 6.8) was added to the mixture followed by the addition of 40 *µ*L of pNPG (4-nitrophenyl-*α*-d-glucopyranoside) as the substrate. The reaction mixture was incubated at 37°C for 15 minutes, and absorbance was measured at 405 nm. The absorbance of the control was recorded from a reaction mixture in which the enzyme was substituted by the same volume of buffer solution. The percentage of enzyme inhibition was calculated by using the following equation:

(2) $$\begin{array}{c} \text{Percentage inhibition}=\frac{\text{Absorbance of control}-\text{Absorbance of sample}}{\text{Absorbance of control}}\times 100. \end{array}$$

The IC_50_ values for plant extracts were calculated by using GraphPad Prism 9 software.

### 2.7. Antibacterial Assay

Antibacterial assay of plant extract was performed by using the agar well diffusion method [25]. Common human pathogens *Klebsiella pneumoniae* (ATCC700603)*, Staphylococcus aureus* (ATCC25923)*, Bacillus subtilis* (ATCC35021), and *Escherichia coli* (ATCC25922) were tested against plant extracts. Overnight incubated broth cultures of test organisms were prepared in nutrient broth media. The concentration of test organisms was maintained at 0.5 McFarland standard (10^6−8^ CFU/mL). About 100 *µ*L of inoculum was taken and spread on MHA agar plates. Then, wells of 7 mm diameter were bored on MHA plates with the help of a sterile cork-borer. 20 *µ*L of plant extract (25 mg/mL in DMSO) was added to the wells in triplicates. The plates were incubated at 37°C for 24 hours. After this, the zone of inhibition (ZOI) was measured using a ruler. 100% DMSO was used as control, and ampicillin (1 mg/mL) was used as standard.

### 2.8. Brine Shrimp Toxicity

A brine shrimp toxicity assay was performed to estimate the lethality of plant extracts. The assay was performed according to standard protocols as described by Meyer et al. [26]. Plant extract solutions of 1000, 100, and 10 *µ*g/mL concentrations in methanol were prepared by the serial dilution of stock solution (10 mg/mL). 2 mL of each concentration was taken in three test tubes for triplicates, and the solvent was evaporated to dryness using a water bath. Equal volume of methanol was also taken in a test tube as a control and evaporated to dryness. The leftover residue was dissolved by adding 5 mL of artificial seawater. 10 healthy brine shrimp larvae (nauplii) were added to each test tube and left for 24 hours, after which the number of surviving nauplii was counted. Thus, the obtained data were used to construct a percentage mortality versus concentration curve. The curve was then used to calculate the concentration of the plant extract lethal to half of the test organisms (LC_50_).

## 3. Results

### 3.1. Phytochemical Analysis

Many important groups of phytochemicals were detected in the extracts of *C. odorata*. Polyphenols, flavonoids, quinones, coumarins, reducing sugar, and terpenoids were observed in all extracts of *C. odorata* (Table 2). CLM, CFM, CSM, and CSA displayed 10 out of 12 phytochemicals screened. CFA and CLA each showed the presence of 9 phytochemicals. Alkaloids were only present in CFM and CLM. The absence of glycosides in CLA, sterols in CFA, and tannins in CSA was observed. Saponins were found only in aqueous extracts.

### 3.2. Total Phenolic and Flavonoid Content

Methanol and aqueous extracts of *C. odorata* contained significant amounts of phenolics and flavonoids (Table 3). CLM displayed the highest TPC and TFC values with 182.26 ± 1.92 mg GAE/g and 128.57 ± 7.62 mg QE/g, respectively. It was followed by that of CFM with TPC and TFC of 172.65 ± 0.48 mg GAE/g and 128.57 ± 7.62 mg QE/g, respectively. The lowest value of TPC was found in CSM (113.6 ± 3.51 mg GAE/g) and that of TFC in CSA (13.14 ± 1.98 mg QE/g). The decreasing order of TPC was CLM > CFM > CFA > CSA > CLA > CSM, whereas the decreasing order of TFC was found to be CLM > CFM > CFA > CLA > CSM > CSA.

### 3.3. Antioxidant Activity

**Figure 2:**
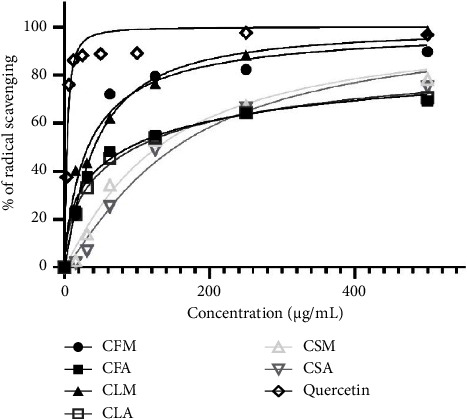
Plot of percentage radical scavenging against concentrations (*µ*g/mL) of plant extracts and standard (values are significantly different from their respective controls at *p* < 0.0001).

**Figure 3:**
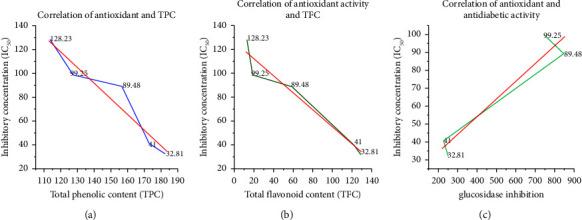
Correlation between (a) TPC and antioxidant, (b) TFC and antioxidant, and (c) antidiabetic and antioxidant activities as IC_50_ (all correlations are significant at *p* < 0.05).

Plant extracts displayed concentration-dependent increments in radical scavenging activities as shown in Figure 2. Methanol extracts showed comparatively higher radical scavenging and lower IC_50_ values than their respective aqueous extracts. Leaf and flower extracts were higher in antioxidant activity than stem extracts. The decreasing order of observed antioxidant activities for different plant extracts is CLM > CFM > CFA > CLA > CSM > CSA (Table 3). This order correlates with the decreasing order of TPC and TFC observed in the study. Extracts having higher concentrations of phenolic and flavonoids showed lower IC_50_ values and thus higher antioxidant activity which is in agreement with the previous reports [27]. Phenolic compounds are known to scavenge free radicals by donating hydrogen atoms from their hydroxyl groups. Among all extracts, CLM was found to contain the highest antioxidant activity as it displayed the lowest IC_50_ value of 32.81 ± 5.26 *µ*g/mL followed by CFM with 41.00 ± 1.10 *µ*g/mL. However, both values were slightly higher than the value of quercetin (3.74 ± 0.15 *µ*g/mL) used as standard. Nevertheless, flower and leaf extracts of *C. odorata* displayed significant radical scavenging activity. CSA displayed the least antioxidant activity with its highest IC_50_ value of 152.93 ± 4.71 *µ*g/mL. The correlation between antioxidant activity and the TPC and TFC content and antidiabetic activity in the plant extracts is shown in Figure 3.

### 3.4. Antidiabetic Activity

**Figure 4:**
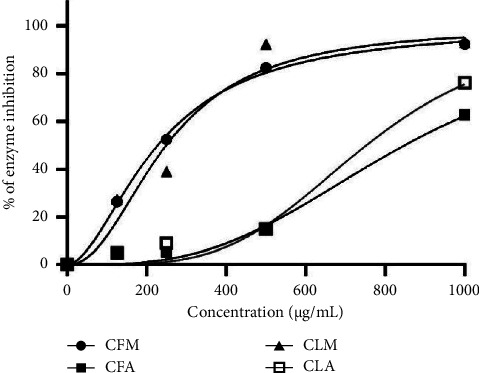
Plot of percentage enzyme inhibition against concentrations (*µ*g/mL) of plant extracts (values are significantly different from their respective controls at *p* < 0.001).

The stem extracts of *C. odorata* did not display significant enzyme inhibition during the screening of *α*-glucosidase inhibition assay and hence were excluded from the determination of IC_50_, but significant antidiabetic activity was observed in flower and leaf extracts of the plant. The increasing order of IC_50_ values was found to be CFM > CLM > CLA > CFA (Table 3). Hence, CFM displayed the highest antidiabetic activity with the lowest IC_50_ value of 227.63 ± 11.38 *µ*g/mL closely followed by CLM with 249.50 ± 5.97 *µ*g/mL. The presence of alkaloids, a high concentration of phenolics, and flavonoids in addition to tannins and terpenoids may be responsible for such higher *α*-glucosidase inhibitory activity in CFM and CLM [28] (Figure 4).

### 3.5. Antibacterial Activity

**Figure 5:**
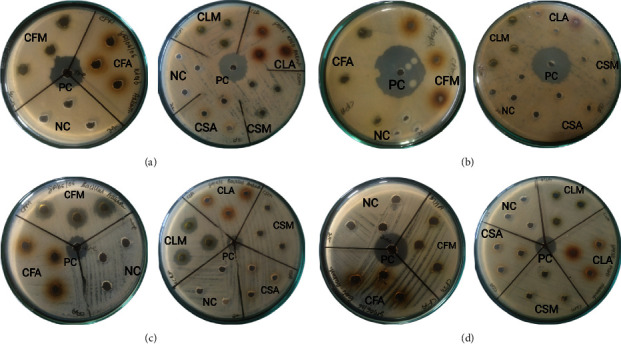
Antibacterial activity (ZOI) of plant extracts against the bacterial strains ((a) against *Klebsiella pneumoniae*, (b) against *Staphylococcus aureus,* (c) against *Bacillus subtilis*, and (d) against *Escherichia coli*); PC, positive control; NC, negative control.

The plant extracts displayed significant activity against Gram-positive (*Bacillus subtilis*) and Gram-negative bacteria (*Klebsiella pneumoniae* and *Escherichia coli*). CLM displayed the most bioactivity as it displayed a significant zone of inhibition against three (*K. pneumoniae, B. subtilis*, and *E. coli*) out of four bacterial strains employed in the assay (Table 4, Figure 5). The highest ZOI (15 ± 0 mm) was displayed against *B. subtilis* and was found to be more potent than ampicillin (14.5 ± 0.48 mm). CFM, CLA, and CSA were found active against two bacteria. CFA was found to be only active against *B. subtilis*, and CSM was found to be active only against *K. pneumoniae*. None of the extracts displayed any significant ZOI against *S. aureus*, whereas *K. pneumoniae* and *B. subtilis* were found to be highly susceptible to *C. odorata*. All the ZOI recorded for plant extracts were found to be less than that recorded for ampicillin except for CLM against *B. subtilis*.

### 3.6. Toxicity

**Figure 6:**
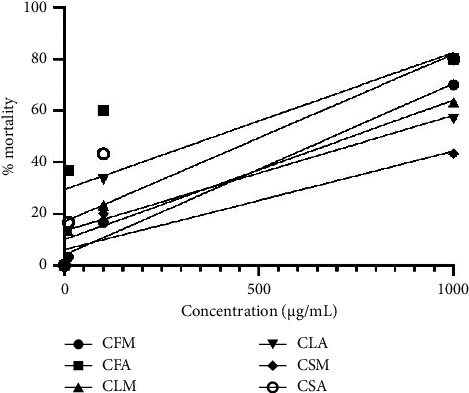
Plot of percentage mortality against concentrations (*µ*g/mL) of the plant extract (values are significantly different from their respective controls at *p* < 0.0001).

The toxicity is reported in decreasing order of LC_50_ for different extracts as CSM > CLA > CLM > CFM > CSA > CFA (Table 5). Extracts having LC_50_ less than 1000 are considered pharmacologically active. All the extracts except CSM showed a toxic effect against brine shrimp nauplii. CFA showed the highest lethality with an LC_50_ of 107.31 ± 49.04 *µ*g/mL, followed by CSA with 470.23 ± 87.08 *µ*g/mL. The aqueous extracts were found to be more toxic than their methanol extract counterparts. The findings of this study suggest that the flower of *C. odorata* is comparatively more toxic than its leaf and stem. The high toxicity in plant extracts can be used in toxic or side effect-based drug discovery strategies (Figure 6).

## 4. Discussion

The presence of phenols, flavonoids, alkaloids, tannins, terpenoids, and coumarins and the absence of saponin in CLM are reported for *C. odorata.* Akinmoladun et al. reported the presence of glycosides in CLM; however, it was reported absent by Mathew et al. [29, 30]. Similarly, the results reported in the literature agreed in the case of alkaloids, saponins, tannins, terpenoids, flavonoids, coumarins, and phenols but did not agree in the case of glycosides and quinones for CLA [30–32]. This may be due to the impacts of altitude, plant growth, environmental stress, harvesting period, and laboratory setup. However, the phytochemical analysis of stem and flower extracts using methanol and the aqueous solvent is the first report in this study. Different phytochemicals are responsible for the bioactivity of plants. Flavonoids, phenols, alkaloids, terpenoids, and coumarins display antioxidant, antidiabetic, anticancer, anti-inflammatory, and antimicrobial activities [33]. Many drugs such as aspirin, digoxin, paclitaxel, artemisinin, and quinine used in modern chemotherapy are isolated or derived from plant sources [34]. The observed prevalence of important phytochemicals in *C. odorata* makes this plant a viable candidate as a source of bioactive compounds in the drug discovery and development process. In recent years, the metabolites in the plant samples can also be analyzed using GC-MS and LC-MS/MS in which the bioactive target metabolites are known by analyzing the mass spectra with the help of the computer software. In this method, a significant peak is detected and the possible fragments and stable ions are analyzed. The metabolomics study part is not included in this paper, but the preliminary phytochemical analysis is included to make the study from preliminary to advanced. The estimation of total phenolics and flavonoids showed that the extracts were rich in these metabolites. The content of phenols and flavonoids was found to be comparatively higher in flowers and leaves than in stems. Similarly, methanol extracts displayed higher content than their aqueous counterparts. Such trends were also reported in the previous research [35]. The TPC in CLM is reported as 455.55 ± 4.59 mg GAE/g by Melinda et al. [36]. In the case of CLA, the TPC was found to be lower than 379.0 ± 7.00 and 198.02 ± 3.96 mg GAE/g reported in the literature [35, 37]. Phenolic contents observed in this study for CLM and CSM were found to be higher than 71.08 ± 0.38 mg GAE/g reported in the literature for the methanol extract of the whole plant [38]. Although there were slight variations in TPC and TFC values in the present studies and the previous literature, they almost agreed with each other. Slight variations in TPC and TFC among previous studies and the present study may have arisen due to several factors including temperature, rainfall, soil composition, and ultraviolet radiation [39]. Flavonoids and phenolic compounds present in *C. odorata* prevent oxidative damage to proteins and cultured skin cells and display cytotoxicity against cancer cells and pharmacological activity against pathogens [40]. In this study, the radical scavenging activity measured in IC_50_ for methanol and aqueous leaf extracts was found to be similar to 0.07 ± 0.003 mg/mL reported for the ethanolic extract of leaves by Omoregie et al. [35]. The antioxidant activity shown by the plant extract is due to the presence of various phytochemicals such as phenols, carotenoids, vitamins, and glucosinolates [41]. A significant correlation between TPC and TFC with antioxidant activity was observed in the present study. The antioxidant activity in plant extracts carries significant importance as it provides protection from oxidative stress, maintains cellular health, supports the immune system, and prevents various cardiovascular and neurodegenerative diseases, diabetes, and cancer [42].

The correlation was also observed between free radical scavenging and antidiabetic activity shown by the plant extracts. Methanol extracts of flowers and leaves displayed significantly higher antidiabetic activity than their corresponding aqueous extracts. Such a trend is also reported in the previous literature [15]. The IC_50_ values of 249.50 ± 5.97 and 752.50 ± 30.88 *µ*g/mL for CLM and CLA, respectively, were slightly lower than 1329.31 ± 2.68 and 1250 *µ*g/mL reported by Putri and Fatmawati [15]. Nevertheless, *C. odorata* contained significant *α*-glucosidase enzyme inhibition activity. Further work in identifying specific metabolites responsible for antidiabetic activity can be accomplished using molecular docking and simulation between reported plant secondary metabolites and enzymes [43]. The bimolecular ligand-protein interaction may also provide insights into the mechanism of action [44]. The ZOI showed by CLM against *K. pneumoniae* and *E. coli* (9.67 ± 0 and 7.3 ± 0.32 mm, respectively) was found comparable to that observed by Natheer et al. (8 and 9 mm, respectively) [45]. Whereas, the ZOI against *S. aureus* and *K. pneumoniae* (0 and 7.3 ± 0.32 mm, respectively) was lower than that observed by Alabi et al. (9.33 ± 0.33 and 16 ± 0.58 mm, respectively) [27]. Similarly, Vijayaraghavan et al. (2018) also observed no ZOI against *E. coli* for CLA [31], whereas ZOI of 11 mm against *B. subtilis* (at 30 mg/mL) was comparable to ZOI of 10 ± 0.57 mm observed in this study [31]. Plant secondary metabolites such as alkaloids, flavonoids, and tannins are reported to have antibacterial properties [46]. Hence, comparatively higher concentrations of flavonoids and the presence of alkaloids in CLM may have contributed to the higher antibacterial activity. The toxicity study showed that LC_50_ value of 830.09 ± 115.57 *µ*g/mL for CLA was higher than 392 *µ*g/mL as reported by Asomugha et al. [47]. Juliani calculated the LC_50_ value from the essential oil of *C. odorata* to be 52.16 *µ*g/mL [48]. The findings of this study suggest that the leaves, stems, and flowers of plants are pharmacologically active. Toxicity in plants may arise due to individual or synergetic interactions of various metabolites. Certain alkaloids, terpenoids, and polyphenols are reported to pose remarkable cytotoxic activities [49]. This high cytotoxicity activity of the plant extract may be responsible for its insecticidal cytotoxic and antimicrobial properties.

## 5. Conclusions

*C. odorata* displays a significant quantity of flavonoids and phenolics showing antioxidant, antidiabetic, antibacterial, and cytotoxic activities. The results showed a correlation between the concentration of phenolics and flavonoids with antioxidant, antidiabetic, and antibacterial activities. The flower and leaf of the plant displayed higher concentrations of phytochemicals and more antioxidant, antibacterial, and antidiabetic activities than the stem extract. Thus, the leaf extract of this plant could be used against bacterial infection which supports the traditional use of this plant against infectious diseases. The methanol extracts were found to be richer in phenolic and flavonoid content and subsequent antioxidant, antimicrobial, and antidiabetic activities than their aqueous extract counterparts. Although the exact cause of such correlation is not known, similar trends were also observed in the literature. The plant extracts displayed potential free radical scavenging and *α*-glucosidase inhibitory activities. The present study authenticates the importance of *C. odorata* as a valuable medicinal plant with substantial pharmacological activity. However, it also necessitates further work on the isolation, purification, characterization, and standardization of bioactive compounds. The findings of this study support the traditional use of *C. odorata* in the control of diabetes as a viable candidate that should be explored for noble therapeutic agents against diabetes.

## Figures and Tables

**Table 1:** Voucher number, plant parts used, and the traditional medicinal uses.

Name of the plant	Identification no.	Altitude/plant growing	Plant parts	Traditional uses
*Chromolaena odorata*	TUCH21052	Altitude 620 m, coordinates 28° 06′ 22″ N, 84° 05′ 13″ E	Flower	Wounds and skin infections [14]
			Stem	Leech bites [18]
			Leaf	Diabetes and soft tissue wounds [15]
				Diarrhoea and malaria [16]

**Table 2:** Qualitative phytochemical analysis of plant extracts.

Group of compounds	CFM	CFA	CLM	CLA	CSM	CSA
Alkaloids	+	−	+	−	−	−
Coumarins	+	+	+	+	+	+
Flavonoids	+	+	+	+	+	+
Glycosides	+	+	+	−	+	+
Polyphenols	+	+	+	_+_	+	+
Quinones	+	+	+	+	+	+
Reducing sugars	+	+	+	+	+	+
Saponins	−	+	−	+	−	+
Terpenoids	+	+	+	+	+	+
Tannins	+	+	+	+	+	−
Proteins	−	−	−	−	+	+
Sterols	+	−	+	+	+	+

CFM, methanol extract of flowers; CFA, aqueous extract of flowers; CLM, methanol extract of leaves; CLA, aqueous extract of leaves; CSM, methanol extract of stems; CSA, aqueous extract of stems; +, present; −, absent.

**Table 3:** TPC, TFC, DPPH free radical scavenging activity (IC_50_), and *α*-glucosidase inhibitory activities of *C. odorata*.

Plant extracts	TPC (mg GAE/g)	TFC (mg QE/g)	IC_50_ (*µ*g/mL)	
			Antioxidant activity	*α*-Glucosidase inhibition
CFM	172.65 ± 0.48	121.74 ± 7.06	41.00 ± 1.10^*∗∗∗*^	227.63 ± 11.38^*∗∗∗*^
CFA	156.26 ± 4.16	58.68 ± 11.36	89.48 ± 3.97^*∗∗∗*^	848.56 ± 19.03^*∗*^
CLM	182.26 ± 1.92	128.57 ± 7.62	32.81 ± 5.26^*∗∗∗*^	249.50 ± 5.97^*∗∗∗*^
CLA	126.65 ± 7.33	18.84 ± 7.89	99.25 ± 1.91^*∗∗∗*^	752.50 ± 30.88^*∗*^
CSM	113.6 ± 3.51	13.14 ± 1.98	128.23 ± 3.03^*∗∗∗*^	—
CSA	132.76 ± 2.77	3.79 ± 2.25	152.93 ± 4.71^*∗∗∗*^	—
^#^Quercetin	**—**	—	3.74 ± 0.15^*∗∗∗*^	—

Values are the mean ± SD (*n* = 3); ^#^positive standard; —, values not measured; values are significantly different from their respective controls at ^*∗*^*p* < 0.05; ^*∗∗∗*^*p* < 0.001.

**Table 4:** Antibacterial activity (ZOI) shown by the plant extracts.

Plant extracts	ZOI (mm) of plant extracts			
	*K. pneumoniae*	*S. aureus*	*B. subtilis*	*E. coli*
CFM	8.66 ± 0.32^*∗∗*^	—	13.33 ± 0.87^*∗∗*^	—
CFA	—	—	7 ± 0^*∗∗*^	—
CLM	9.67 ± 0.32^*∗∗*^	—	15 ± 0^*∗∗*^	7.3 ± 0.32^*∗∗*^
CLA	11 ± 0.99^*∗∗*^	—	10 ± 0.57^*∗∗*^	—
CSM	8.33 ± 0.66^*∗∗*^	—	—	—
CSA	8.6 ± 0.32^*∗∗*^	—	9.3 ± 0.32^*∗∗*^	—
^#^Ampicillin	24 ± 1.68^*∗*^	35 ± 1.18^*∗*^	14.5 ± 0.84^*∗*^	22 ± 0^*∗*^

Values are the mean ± SE (*n* = 3); ^#^positive standard; —, no significant ZOI; values are significantly different from their respective controls at ^*∗*^*p* < 0.05; ^*∗∗*^*p* < 0.01.

**Table 5:** Concentration lethal to 50% of test organisms (LC_50_) shown by plant extracts against brine shrimp larvae.

Plant extracts	LC_50_ (*µ*g/mL)
CFM	691.91 ± 71.46^*∗*^
CFA	107.31 ± 49.04^*∗∗∗*^
CLM	711.97 ± 77.25^*∗∗*^
CLA	830.09 ± 115.57^*∗∗∗*^
CSM	1204.05 ± 191.28^*∗∗*^
CSA	470.23 ± 87.08^*∗∗*^

Values are the mean ± SD (*n* = 3); values are significantly different from their respective controls at ^*∗*^*p* < 0.05; ^*∗∗*^*p* < 0.01; ^*∗∗∗*^*p* < 0.001.

## Data Availability

The data used to support the findings of the study are available from the corresponding author upon request.
